# Thrombus in Transit: Key Echocardiography Findings in the ED

**DOI:** 10.7759/cureus.69109

**Published:** 2024-09-10

**Authors:** Leah Flanagan, Petrus DuPlooy, Gillian Judge, Cian McDermott

**Affiliations:** 1 Emergency Medicine, Mater Misericordiae University Hospital, Dublin, IRL

**Keywords:** acute pulmonary embolism, clot in transit, emergency echocardiography, interventional radiology techniques, right atrial clot

## Abstract

A 32-year-old Asian male presented to the ED with a one-day history of mild pleuritic chest pain. He was diagnosed with an acute pulmonary embolus on CT Pulmonary Angiography (CT-PA). Transthoracic echocardiography (TTE) performed at the bedside in the ED demonstrated evidence of right heart strain but, most notably, a highly mobile echogenic thrombus in the right atrium, consistent with a clot-in-transit (CIT). This was not visualized on CT due to the influx of contrast in the heart. Based on this, the patient was transferred to the High Dependency Unit for IV heparin and close monitoring. The following day, he underwent clot retrieval using an Inari Flowtriever under direct TTE guidance. He was discharged on oral anticoagulation four days later and experienced no complications on follow-up. CIT is an important feature of pulmonary embolus to identify, as it can escalate the risk stratification of the patient, and management will need to be altered accordingly.

## Introduction

Venous thromboembolism (VTE), which includes both deep vein thrombosis (DVT) and pulmonary embolism (PE), is a major contributor to cardiovascular morbidity and mortality worldwide. In Western countries, PE is the third leading cause of cardiovascular-related deaths, with its prevalence expected to increase over time in the context of an aging population [1]. Despite advancements in diagnostic techniques and treatment options, PE retains a mortality rate of 5-7%, with a substantial risk of underdiagnosis and undertreatment [2,3].

Effective risk stratification of PE at the time of presentation is crucial but remains challenging. PE can present with a wide spectrum of clinical manifestations, ranging from mild symptoms to sudden cardiac arrest, making it difficult to accurately assess the severity and risk in each patient. The condition may also present asymptomatically or without obvious hemodynamic compromise, further complicating timely diagnosis. Nearly half of all PEs are diagnosed in emergency settings, where time-sensitive decision-making is essential [2].

Transthoracic echocardiography (TTE) has emerged as a valuable tool in this context, allowing trained clinicians to quickly identify high-risk features, such as right atrial thrombus or clot-in-transit (CIT), which are associated with an increased risk of adverse outcomes [3]. CIT is a specific, rare phenomenon where a thromboembolism temporarily lodges in the right heart before moving through the low-pressure pulmonary vasculature, where it can rapidly cause a life-threatening PE [4-6]. This has been shown to have a worse 30-day outcome and a three-fold increase in mortality in patients with intermediate-risk PE and right ventricular dysfunction (RVD) [2,4].

RVD or 'right heart strain', in the correct clinical context, can be indicative of increased pulmonary vascular resistance leading to increased right ventricular afterload as it strains to contract against the pulmonary artery. PE can cause RV dilatation and dysfunction because of this pattern, leading to RV failure and subsequently, death [7].

Systematic reviews and meta-analyses have shown an increase in short-term mortality if this is demonstrated on TTE in patients who are hemodynamically stable. However, the European Society of Cardiology (ESC) Guidelines (2019) still do not routinely recommend echocardiography in the risk stratification of patients as it has suggested that previously, it would not be used to alter patient management. This case report highlights the necessity of TTE in the risk stratification and management of PE.

We present the case of a 32-year-old Asian male who arrived at the ED with acute onset of pleuritic chest pain and dyspnea. Initial clinical assessment revealed signs of right heart strain, with further imaging confirming a large volume bilateral PE and a right atrial thrombus, a rare and potentially fatal phenomenon known as CIT. This case underscores the critical role of timely diagnostic imaging and intervention in managing high-risk PE cases complicated by CIT. TTE was instrumental in diagnosing the CIT, as the right atrial thrombus was obscured by contrast on the CT scan, underscoring the necessity of TTE in detecting such critical findings that may be missed on cross-sectional imaging.

## Case presentation

History

A 32-year-old Asian male presented to the ED with a one-day history of mild pleuritic chest pain and significant dyspnea. He had no preceding fever, cough, hemoptysis, or lower limb swelling. Apart from an initial diagnosis of “pleurisy” and a lower respiratory tract infection with hemoptysis, there was no medical history of note. He led a sedentary lifestyle as an overnight security guard.

Examination

Physical examination revealed sinus tachycardia of 112 bpm and an oxygen saturation of 95% on room air. Systolic blood pressure (SBP) was 100 mmHg, and his respiratory rate was 16 breaths per minute. Lung fields were clear on auscultation. Lower limbs were soft and supple, without clinical suspicion for deep venous thrombosis (DVT).

Laboratory results

Table 1 demonstrates the laboratory results that became available. Serum troponin, D-dimer, and proNT-BNP levels were elevated at 105 ng/L, 0.99 mg/L, and 745 ng/L respectively. These cardiac biomarkers are important in the risk stratification of PE as outlined in the ESC Guidelines [2].

**Table 1:** Laboratory values demonstrating elevated cardiac biomarkers.

	Lab value	Normal range
Troponin (hsTNI)	105 ng/L	(<34 ng/L)
Pro N-Terminal B-Type Natriuretic Peptide (proNT-BNP)	745 ng/L	(<300 ng/L)
D-Dimer	0.99 mg/L	(0.00-0.50 mg/L)

Radiological imaging

**Figure 1:**
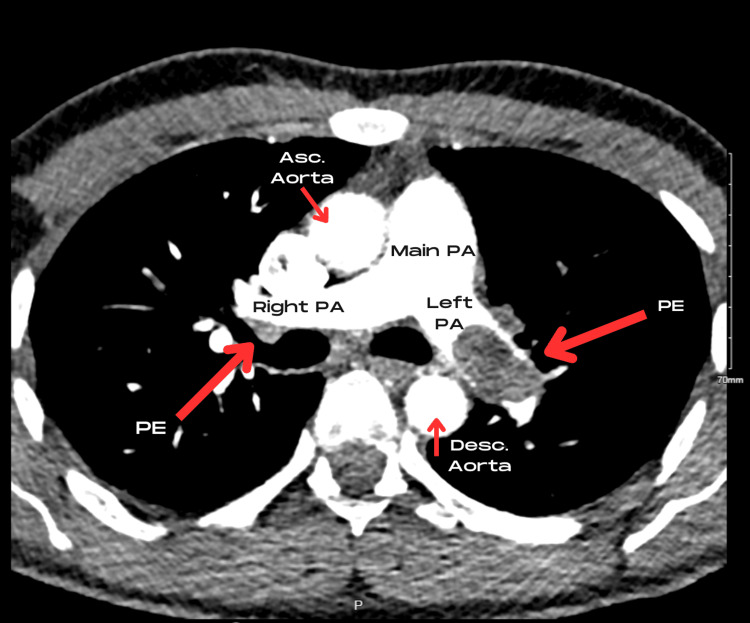
Bilateral pulmonary emboli visualized on CT pulmonary angiogram. PE: Pulmonary Embolism; PA: Pulmonary Artery; Asc. Aorta: Ascending Aorta; Desc. Aorta: Descending Aorta.

**Figure 2:**
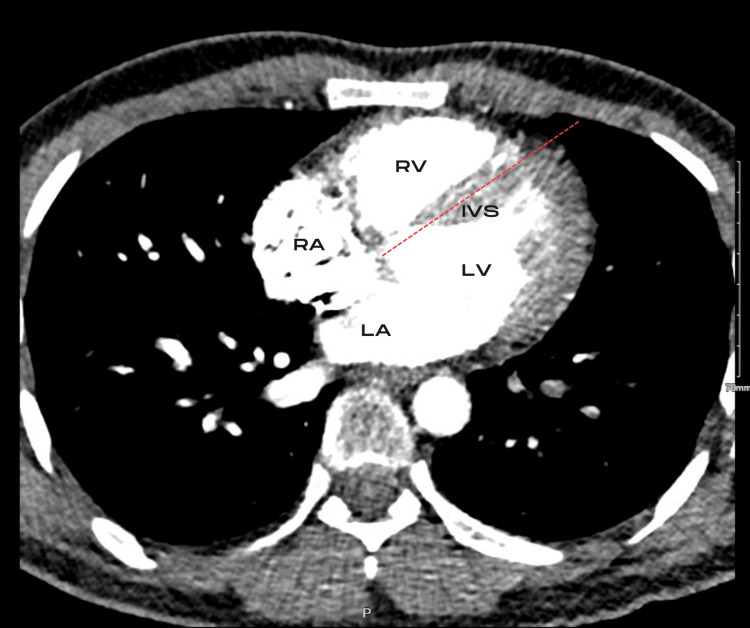
Evidence of right heart strain with flattening of the interventricular septum on CT pulmonary angiogram. RV: Right Ventricle; RA: Right Atrium; LV: Left Ventricle; LA: Left Atrium; IVS: Interventricular Septum.

The decision was made to proceed to CT Pulmonary Angiogram (CT-PA) based on a Wells score of 1 [8], with a positive D-dimer and a negative chest X-ray. This showed large-volume bilateral central pulmonary artery emboli resulting in occlusion of the left upper lobe pulmonary artery and near-complete occlusion of the left lower lobe pulmonary artery (Figure 1). The arrow in Figure 1 shows a large filling defect in the left pulmonary artery and, similarly, a smaller region of thrombus in the right pulmonary artery. Importantly, there was radiological evidence of right heart strain with a flattened interventricular septum as demonstrated by the dotted line in Figure 2. Small foci of consolidation were noted in the right lower lobe, potentially representing small pulmonary infarctions. Due to the influx of contrast obscuring the view, no mass was seen in the right ventricle (RV) or right atrium (RA) cavity on cross-sectional imaging.

Transthoracic echocardiography

**Figure 3:**
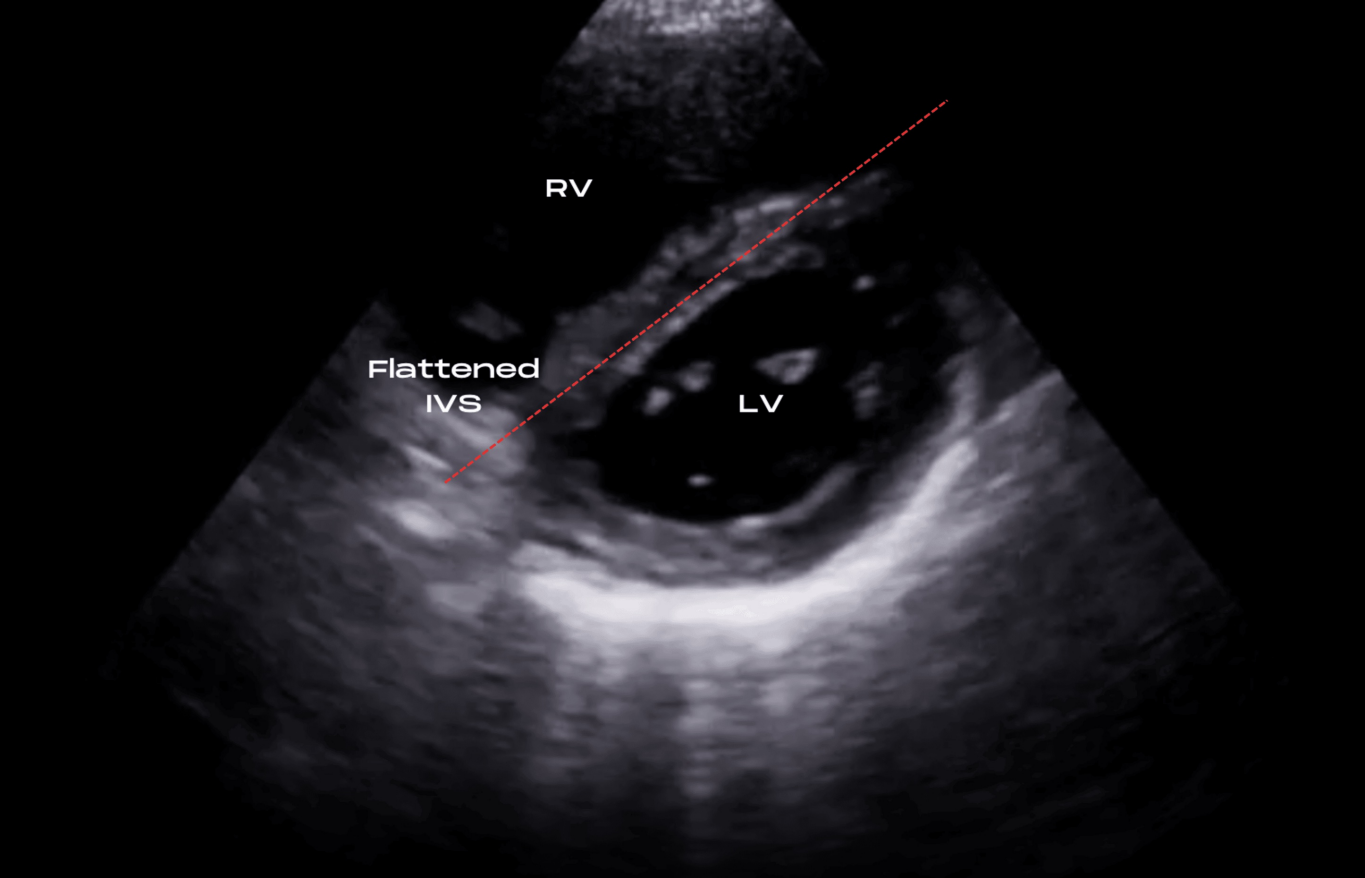
PSAX view with flattening of the interventricular septum, also known as the 'D sign,' indicative of raised RV pressure. PSAX: Parasternal Short Axis View; IVS: Interventricular Septum; RV: Right Ventricle; LV: Left Ventricle.

**Figure 4:**
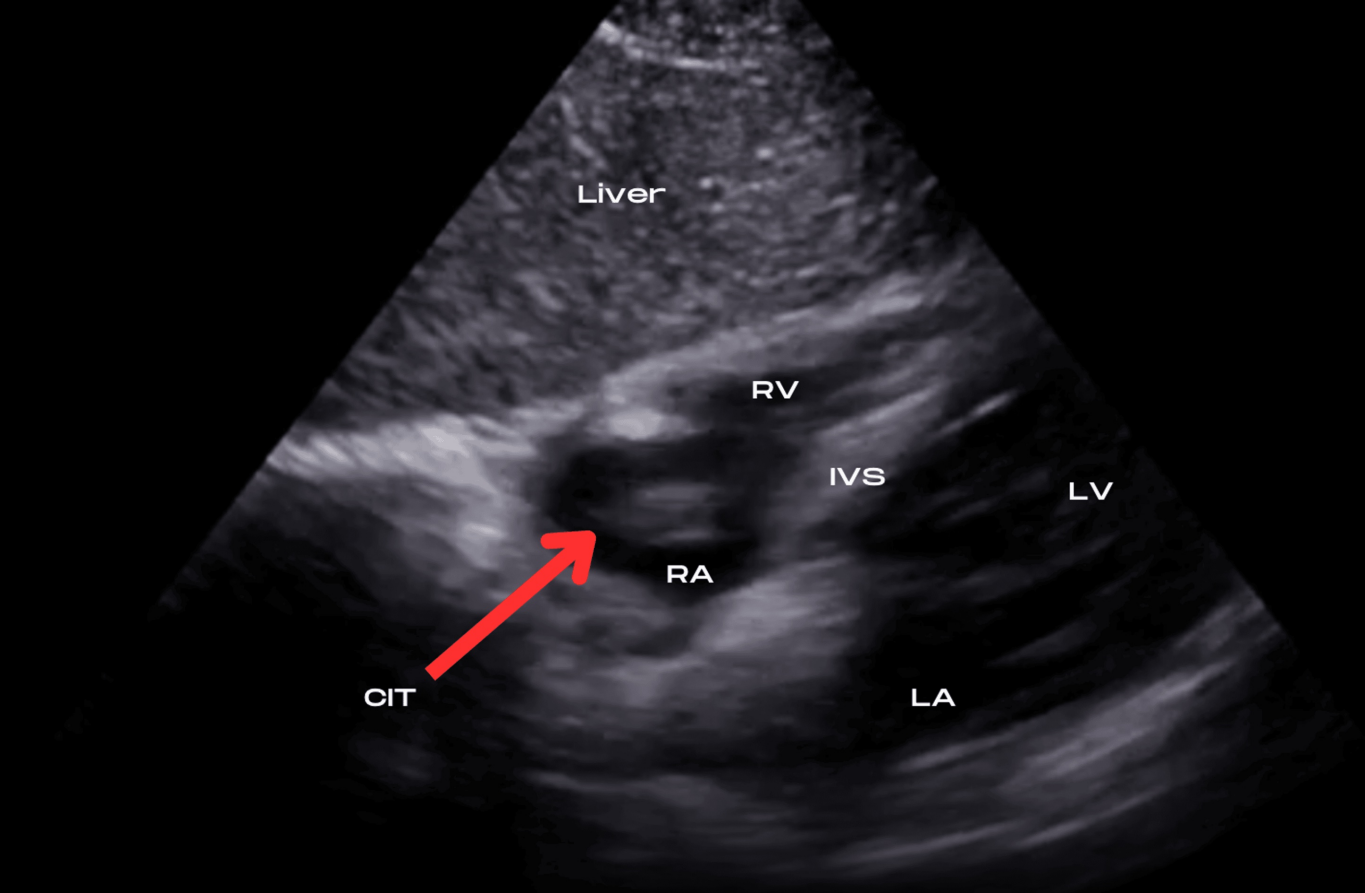
Subcostal view with an echogenic thrombus depicted in the right atrium. CIT: Clot-in-Transit; RV: Right Ventricle; RA: Right Atrium; IVS: Interventricular Septum; LV: Left Ventricle; LA: Left Atrium.

**Figure 5:**
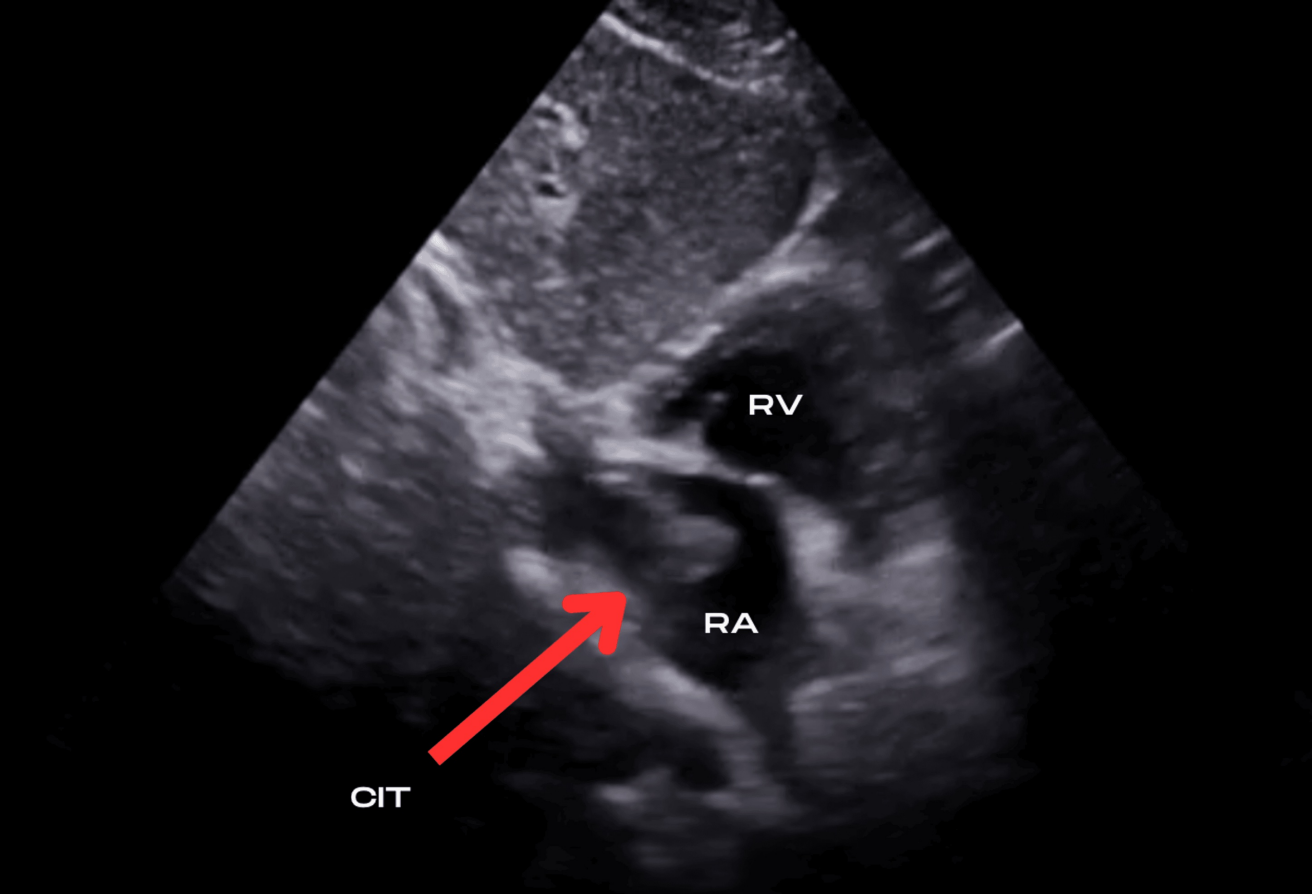
Subcostal right atrium focused view of the thrombus, obtained when rotating to locate the IVC. RV: Right Ventricle; RA: Right Atrium; CIT: Clot-in-Transit; IVC: Inferior Vena Cava.

**Figure 6:**
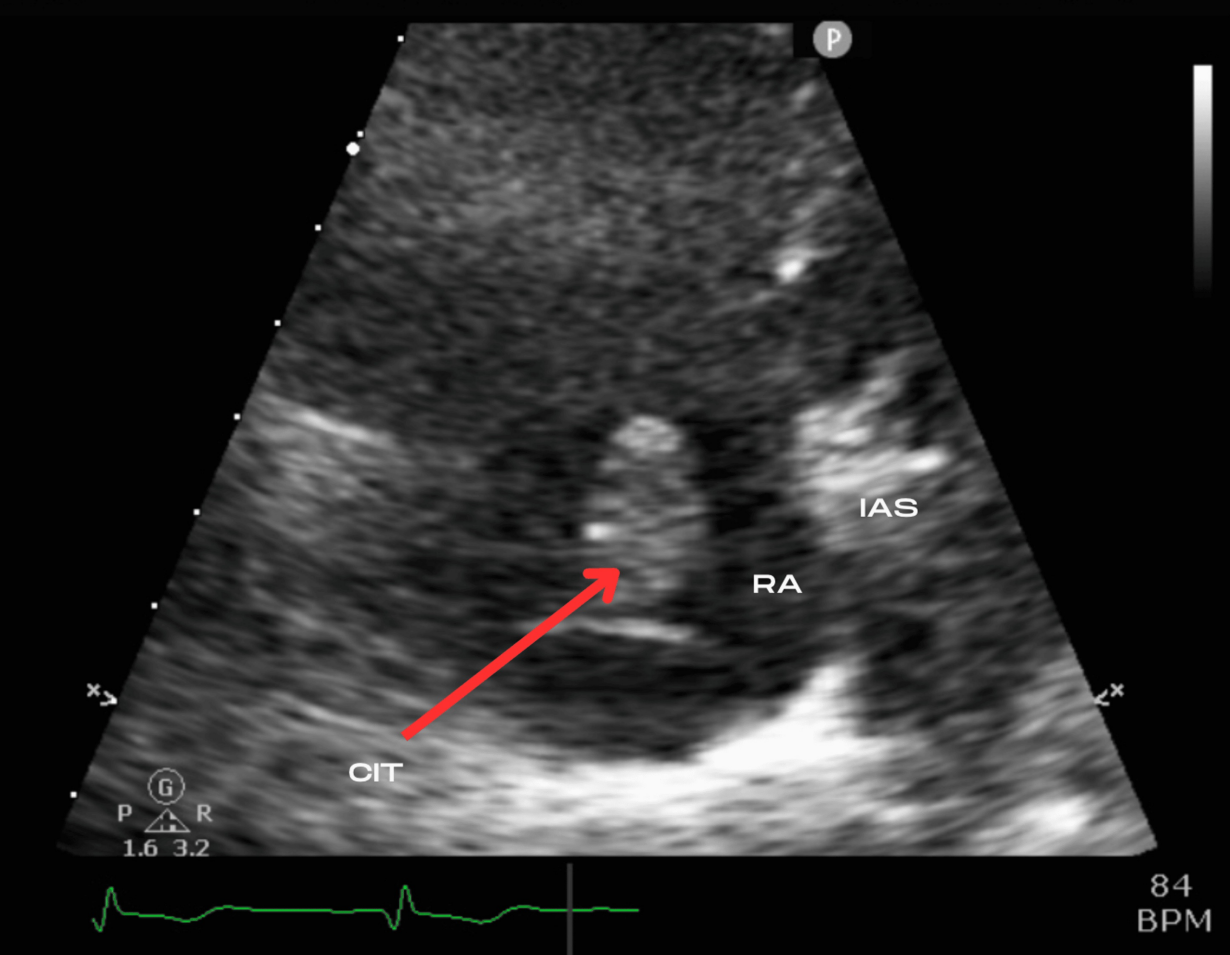
Right atrium focused view from the apex demonstrating a mobile, hyperechoic mass non-adherent to the tricuspid valve. RA: Right Atrium; IAS: Interatrial Septum.

TTE was performed in the ED to evaluate further signs of right heart strain. In static images, this is easily visualized on a parasternal short axis (PSAX) view as seen in Figure 3 with flattening of the interventricular septum in the classic “D-sign”. A mobile echobright lesion was noted in the right atrial chamber, most easily depicted by the arrows on subcostal views as seen in Figures 4-5. This was not adherent to the tricuspid valve structure and was thought to represent a right atrial thrombus. Spectral Doppler findings were consistent with elevated pulmonary vascular resistance and included a dilated RV (5.6 cm basal diameter), McConnell’s sign [8], shortened pulmonary acceleration time, and early-systolic notching of the right ventricular outflow tract Doppler envelope. Longitudinal and radial function of the RV was preserved. Figure 6 focuses further on the RA from an apical viewpoint to characterize the thrombus.

The above clinical findings generated a Pulmonary Embolism Severity Index (PESI) score of Class I (54 points) [9]. Thus, as per the European Society of Cardiology (2019) guidelines [2], a diagnosis of intermediate-high risk PE with an RA thrombus was reached.

Management and outcome

The decision was made to anticoagulate the patient with intravenous heparin and monitor him in the high dependency unit, considering the risk of propagation of the thrombus-in-transit. The right atrial clot was aspirated the following day by the interventional radiology team using an Inari Flowtriever device under direct procedural ultrasound guidance. The patient continued to improve and was transitioned from unfractionated heparin (UFH) to a direct oral anticoagulant (DOAC) upon hospital discharge.

## Discussion

Pulmonary embolism (PE) is one of the leading causes of cardiovascular mortality worldwide [1]. Clot-in-transit (CIT) is a specific, rare phenomenon where thromboembolism temporarily lodges in the right heart before moving through the low-pressure pulmonary vasculature. It is documented in studies as being rare, with a prevalence of between 3-18% in patients presenting with acute PE [4-6]. CIT can also be referred to as right heart thrombi (RHT) and can be visualized at any point in the right atrium, right ventricle, and inferior vena cava (IVC) [10]. This can also be visualized in patients with a patent foramen ovale (PFO) [11]. The prevalence of RHT increases in patients with an increased PESI score [2].

In cases where CIT is diagnosed, patients may be more hemodynamically compromised and have a more acute symptom onset [4,6]. Patients with RHT diagnosed on TTE are also twice as likely to present with congestive heart failure and have evidence of right ventricular hypokinesis [4,6]. Overall mortality rates are also twice as high in patients diagnosed with CIT [10,11].

Three types of CIT have been described in the literature. TEE is the gold standard method of choice for characterizing these thrombi [11,12]. However, patients can be at high risk of deterioration if undergoing sedation or general anesthesia, and as such, features can attempt to be identified on TTE. Type A is a mobile RHT which has a high risk of dislodging and causing rapid hemodynamic decompensation. It is identified by a thin stalk. Type B is an immobile, ovoid-shaped thrombus that is more adherent to the wall of the chamber with a wider base. Type C has characteristics of the two [10]. It is important to note that in this case, the RA mass was obscured by the contrast bolus used on cross-sectional imaging. Thus, it was only possible to identify the CIT by using echocardiography.

TTE, in this case, also identified features of RVD. Systematic reviews and meta-analyses have demonstrated over a two-fold increase in mortality in even hemodynamically stable patients with TTE findings of RVD [7]. However, TTE findings for PE have a low negative predictive value and thus echocardiography should not be used in isolation to exclude this diagnosis [12]. A combination of features used to identify RV strain, however, have a higher specificity for PE and many of these features were noted in this patient in particular. A systematic review published by Fields JM et al. in 2017 demonstrated that RV enlargement demonstrated by physicians was 46% sensitive and 91% specific for PE [3]. This is described as a basal right ventricular diameter of >41mm and mid-level right ventricular diameter >35mm which can be best viewed in an RV-focused apical four-chamber (AP4) view [13].

Often, it can be difficult to ascertain the acuity of RV strain findings, and thus, evaluation of RV wall hypertrophy below the level of the tricuspid annulus can help determine if the phenomenon is chronic [14]. International standardized values for RV diameter can also vary between institutions such as the British Society of Echocardiography (BSE) and American Society of Echocardiography (ASE) [15,16].

The 'McConnell sign,' first described in 1996, is recognized as global hypokinesis of the RV but with paradoxical intact movement at the apex during systole. This has a specificity for PE of 94% [17]. Increased RV and pulmonary circuit pressures, as may be observed in PE, lead to a flattened appearance of the interventricular septum (IVS). On account of interventricular dependence, the IVS appears to bow towards the left at end-diastole, creating an eccentric 'D-shaped' LV on TTE parasternal short axis view (PSAX) as seen in Figure 3 [3]. This finding is also recognized as septal dyskinesia and has a specificity of 96% for PE [12]. A reduced tricuspid annular plane systolic excursion (TAPSE) of ≤ 16mm has also been shown to be an independent predictor of PE-related mortality [2]. An RV/LV ratio of > 1.0 is indicative of RV dilation and has a high specificity of 98% for PE [18]. Mobile thrombi, i.e., CIT noted in the SVC, IVC, RA, RV, or pulmonary arteries, has a specificity of 100% for PE [12].

A variety of different management strategies are available for patients diagnosed with CIT, from anticoagulation and systemic thrombolysis to surgical embolectomy and catheter-directed therapies such as these [2,10,19]. As such, the utilization of TTE to identify these lesions is important. Several studies have shown an increase in mortality in patients with CIT where anticoagulation has been used alone [4,10,19], suggesting that we may need to be more aggressive with our treatment of patients with CIT as opposed to intermediate-high risk PE alone. The significant number of contraindications with systemic thrombolysis and surgical options has led to the rise of endovascular techniques such as that employed in this case [2,20]. The Inari Flowtriever catheter (Inari Medical, Irvine, CA, USA) is a flexible aspiration device used by interventional radiology suites under TEE (or TTE in this case) and has been granted approval for use in CIT and acute PE/DVT by the US FDA.

In this patient’s case, the use of TTE was essential for the diagnosis of CIT, subsequently changing management resulting in a successful patient outcome. Similar results have been demonstrated in other case studies [20]. As has been noted in previous registries, TTE is not always performed in patients with VTE [4]. The 2019 ESC Guidelines recommend its use in patients with suspected high-risk PE who are hemodynamically unstable, but it is not mandated in patients' diagnostic workup when considered hemodynamically stable [2]. However, this case does support the use of TTE in the evaluation of patients who present with intermediate-high or high-risk PE.

## Conclusions

This case illustrates the complexities of diagnosing and managing pulmonary embolism complicated by right atrial thrombus. The use of TTE was pivotal in identifying the CIT, which altered the clinical management strategy, leading to the successful aspiration of the thrombus using an endovascular approach. Given the high risk of deterioration associated with CIT, prompt recognition and intervention are crucial to improving patient outcomes. The case also highlights the potential underuse of echocardiography in evaluating patients with suspected PE, suggesting that broader application of this imaging modality could enhance the detection of evidence of RVS and complications such as CIT, particularly in patients presenting with intermediate to high-risk features.

In conclusion, the successful management of this patient emphasizes the importance of a multidisciplinary approach, incorporating advanced imaging techniques and interventional radiology, to address the challenges posed by complex cases of pulmonary embolism. The evolving landscape of PE treatment offers new avenues for improving survival rates and reducing complications in high-risk patients. This case contributes to the growing body of evidence supporting more aggressive management strategies in the presence of CIT, advocating for the integration of advanced diagnostic and therapeutic modalities, including TTE, in routine clinical practice.
